# Repetitive transcranial magnetic stimulation for treating post-stroke depression: a systematic review and network meta-analysis

**DOI:** 10.1186/s12888-025-07151-1

**Published:** 2025-07-15

**Authors:** Yueying Wang, Huiyue Feng, Hongxia Wang

**Affiliations:** 1 Department of Medicine, Shandong Xiandai University, Jinan, China; 2 Department of Rehabilitation Medicine, The Second Affiliated Hospital of Shandong University of Traditional Chinese Medicine, Jinan, China

**Keywords:** Repetitive transcranial magnetic stimulation, Post-stroke depression, Systematic review, Network meta-analysis

## Abstract

**Objective:**

To systematically evaluate the effects of different modes of repetitive transcranial magnetic stimulation (rTMS) on depression in post-stroke depression (PSD) patients using the network meta-analysis method.

**Methods:**

PubMed, Web of Science, Cochrane Library, Embase, Wanfang Data, CNKI, VIP, and CBM were searched for randomized controlled trials on rTMS for PSD from the time the database was constructed until April 20th, 2024. Two researchers independently conducted screenings to extract the relevant literature. The meta-analysis was performed using RevMan 5.4.1 software. Network meta-analysis of depression scores was performed using Stata 17.0 software and R 4.4.1 software.

**Results:**

Twelve studies involving four different rTMS modalities were included. The results of the meta-analysis revealed that rTMS significantly improved depression (SMD = -1.47, 95% CI [-1.97, -0.97]) and activity of daily living (ADL) (SMD = 0.78, 95% CI [0.52, 1.04]) in patients with PSD. The follow-up results revealed that rTMS significantly improved depression in the long term (SMD = -1.74, 95% CI [-2.32, -1.17]). The results of the network meta-analysis revealed that dual-rTMS (SMD = -2.33, 95% CI [-3.16, -1.50]), HF-rTMS (SMD = -1.62, 95% CI [-2.11, -1.13]), iTBS (SMD = -1.57, 95% CI [-2.56, -0.57]), and LF-rTMS (SMD = -0.80, 95% CI [-1.36, -0.23]) significantly improved depression compared with sham stimulation. The SUCRA values in descending order were dual-rTMS (95.9%) > HF-rTMS (65.0%) > iTBS (61.9%) > LF-rTMS (27.1%).

**Conclusions:**

Twenty minutes of 4-week dual-rTMS on the DLPFC of PSD patients may produce better therapeutic effects. High-quality, large-sample, standardized RCT trials can be conducted to further test the scientific validity of the findings.

**Supplementary Information:**

The online version contains supplementary material available at 10.1186/s12888-025-07151-1.

## Introduction

Post-stroke depression (PSD) is one of the most common sequelae after stroke and manifests as a series of significant and persistent affective disorder syndromes characterized by depressed mood, negative pessimism, and loss of interest [1]. Studies have shown that the prevalence of PSD can reach 33% [2, 3]. PSD can adversely affect the recovery of patients with cerebral infarction, including delaying the recovery of motor function, exacerbating cognitive dysfunction, and even increasing the risk of suicide. The treatment options chosen for PSD in clinical practice are mostly 5-hydroxytryptamine (5-HT) reuptake inhibitor antidepressants [4–6]. However, stroke patients have poor compliance due to the large number of oral medications used, resulting in poor treatment efficacy [7].

Repetitive transcranial magnetic stimulation (rTMS) is a novel neurophysiological stimulation technique that has been used in the rehabilitation of PSD [8]. rTMS can produce brief, rapidly changing magnetic fields [9, 10]. rTMS can increase cortical monoamine transmitter release, increase glucose metabolism, and increase the level of brain-derived neurotrophic factor (BDNF), which is effective in depression [11]. Currently, there are four main rTMS modalities used for the treatment of PSD: high frequency-rTMS (HF-rTMS) (> 1 Hz), low frequency-rTMS (LF-rTMS) (≤ 1 Hz), theta-burst stimulation (TBS), and dual-rTMS [12–14]. Most of the studies focused only on magnetic stimulation of a single cerebral hemisphere. Dual-rTMS refers to the simultaneous action of HF-rTMS and LF-rTMS on both cerebral hemispheres of a patient.

Clinical studies have demonstrated the efficacy of rTMS in the treatment of PSD, but the optimal stimulation pattern of rTMS for PSD is inconclusive. Therefore, this study used a network meta-analysis to compare the effects of different frequencies of rTMS on depression in patients with PSD. This study provides an evidence-based foundation for the selection of clinical rTMS modalities.

## Methods

### Study design and registration

This study followed the Preferred Reporting Items for Systematic Review and Meta-Analysis (PRISMA) guidelines [15] (supplementary file) and is registered in the International Prospective Register of Systematic Reviews (PROSPERO; ID: CRD42024536962).

### Inclusion criteria

#### Study type

Randomized controlled trials (RCTs) limited to Chinese and English.

#### Study population

Patients with a clear clinical diagnosis of PSD, regardless of gender, age, or race.

#### Interventions

rTMS and conventional treatment in the experimental group, sham rTMS (coil using a special angle or using a special sham coil) and conventional treatment in the control group.

#### Outcome indicators

Primary outcome indicators were depression assessment scales, including the Hamilton Depression Rating Scale (HAMD, HDRS), Beck Depression Inventory (BDI), Quick Inventory of Depressive Symptomatology (QIDS), and Montgomery Asberg Depression Rating Scale (MADRS); the secondary outcome indicators were the assessment scales of activities of daily living ability, including the Modified Barthel Index (MBI), Barthel Index (BI), and Functional Independence Measure (FIM).

### Exclusion criteria

The literature exclusion criteria were as follows: (1) reviews, case reports, or conference reports; (2) animal experiments or individual case reports; (3) duplicate publications; and (4) studies with incomplete data or unavailable outcome indicators.

### Search strategy

PubMed, Web of Science, Cochrane Library, Embase, Wanfang Data, CNKI, VIP, and CBM were searched for RCT studies on rTMS for PSD, and the search was conducted from the time of database construction to April 2024. Using PubMed as an example, the search formula was as follows: ((transcranial magnetic stimulation [MeSH] OR TMS OR repetitive transcranial magnetic stimulation OR rTMS OR theta burst stimulation OR TBS) AND (stroke [MeSH] OR post-stroke OR apoplexy OR infarction OR haemorrhage OR cerebrovascular disorder OR cerebrovascular accident OR CVA)) AND (depression [MeSH] OR depressive disorder OR mood disorder OR affective disorder).

### Data extraction

The search results of the databases were imported into the literature management software EndNote 20, and two researchers independently conducted screenings to extract relevant studies that met the inclusion and exclusion criteria. The included studies were cross-checked if the opinions were not unanimous; then, a third, more experienced researcher made the final decision. The following data were extracted: (1) first author and year of publication; (2) rTMS stimulation parameters and period; and (3) outcome indicators.

### Quality assessment

The included studies were evaluated by two investigators following the Cochrane Handbook of Systematic Review in terms of six aspects [16]. If the conditions were fully satisfied, the study was graded as A, indicating low risk; if the study was not mentioned in the text or was mentioned but could not be confirmed, the study was graded as B, indicating medium risk; if the study did not satisfy the conditions, the study was graded as C, indicating high risk.

### Statistical analysis

#### Standard meta-analysis

The meta-analysis was performed using RevMan 5.4.1 software, and forest plots were drawn. Continuous variables were expressed as effect sizes using standard mean difference (SMD), and a 95% confidence interval (CI) was calculated. The results were considered significantly different at *P* < 0.05. The assessment of the results between the included studies was performed; if *I*^2^ ≤ 50% and *P* ≥ 0.1, heterogeneity between studies was considered low and analysed by a fixed-effects model; if *I*^2^ > 50% and *P* < 0.1, heterogeneity was considered to be high and analysed by a random-effects model.

#### Network meta-analysis

The network meta-analysis of depression scores was conducted based on the frequentist approach using Stata 17.0 software and R 4.4.1 software. A network relationship plot was drawn. If no closed loop is formed in the graph, the consistency model is directly used for analysis. If there is a closed loop in the graph, the consistency of the closed loop is assessed via the inconsistency test. If the 95% CI of the inconsistency factor (IF) is 0, the consistency between direct and indirect evidence is good and can be analysed using the consistency model. Conversely, the inconsistency model was used for analysis. A league table of pairwise meta-analyses is produced. The strengths and weaknesses of each intervention were assessed based on the surface under the cumulative ranking curve (SUCRA), which ranges from 0 to 100%. A larger SUCRA indicates a lower depression score and a more favourable regimen for the patient.

#### Additional analysis

To explore the source of heterogeneity, subgroup analysis was performed for depression, and patients were divided into subgroups according to frequency of rTMS stimulation, MT, and degree of depression. Sensitivity analysis was conducted by adopting a one-by-one exclusion method to assess the robustness of the findings. The presence of publication bias was determined by plotting a comparative correction funnel plot and performing Egger’s test [17]. The results were analysed, with *P* < 0.05 indicating a statistically significant difference.

## Results

### Selection of studies and characteristics

**Fig. 1:**
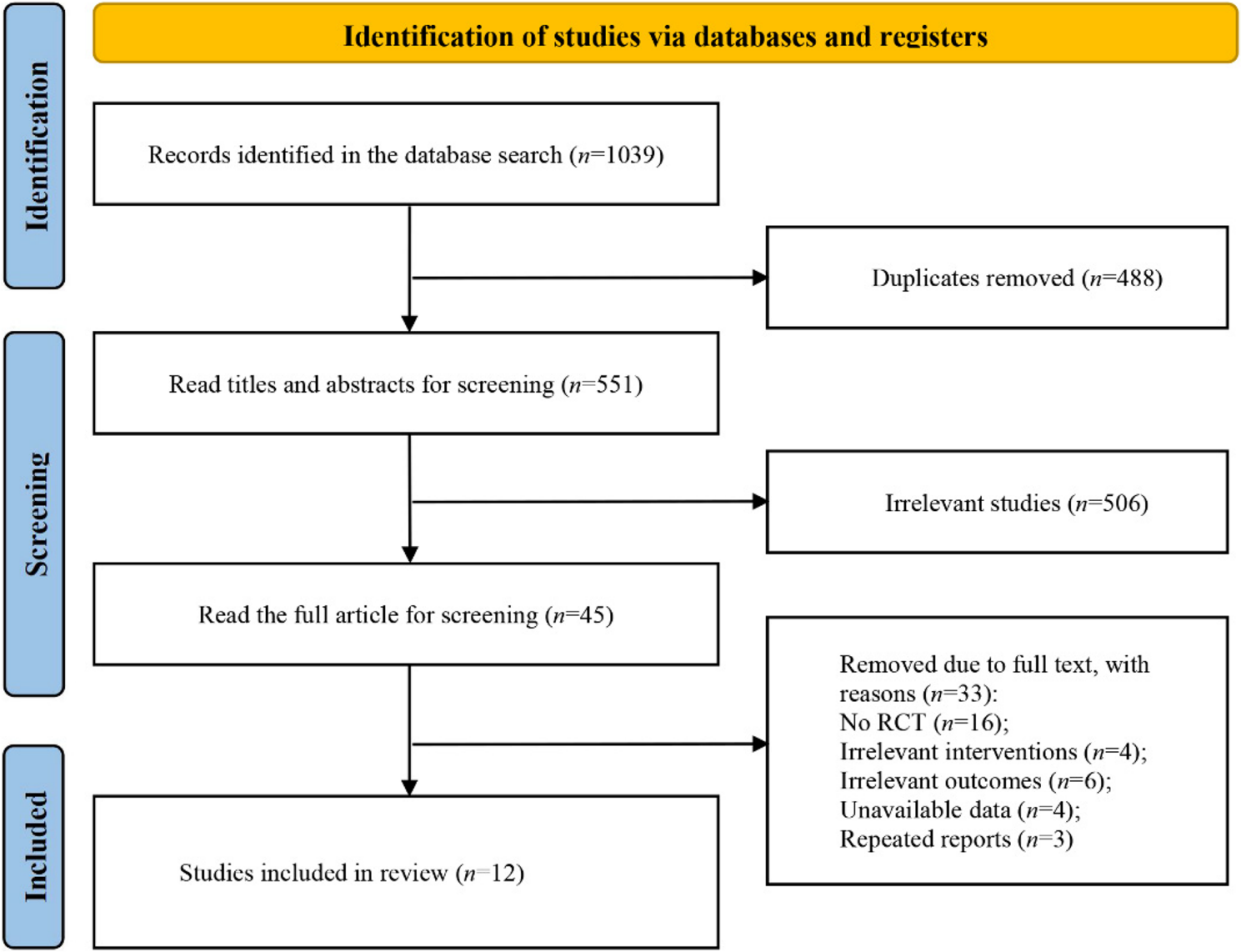
PRISMA flowchart of the screened and included studies

The search resulted in 1039 relevant studies, and 551 studies were obtained via EndNote 20 and manual screening to remove duplicates. The initial screening by reading the title and abstract yielded 45 studies. Twelve studies were included after the full texts were read [18–29]. Interventions included dual-rTMS (2 studies), HF-rTMS (9 studies), LF-rTMS (5 studies), iTBS (2 studies), and Sham (9 studies). The flowchart of the literature screening is shown in Fig. 1. The basic characteristics of the included studies are shown in Table 1.

**Table 1:** Characteristics of the 12 included studies

Author (year)	Country	Sample size (E/C)	Gender (male/female)	Stroke type (infarction/haemorrhage)	Stroke location (right/left/multiple)	Post-stroke duration (days/weeks/ months/years)	Depression level	Age (E/C, year)	Intervention (E/C)	Site of stimulation	rTMS Parameter			Treatment duration (day/week)	Outcome
											MT% TMS model	Train duration/Pause duration	Total pulses		
Kim et al.(2010) [18]	Korea	6/6/6	4/2; 2/4; 4/2	4/2; 5/1; 5/1	3/2/1; 3/2/1; 6/0/0	(241.2 ± 42.5)/ (404.4 ± 71.7)/ (69.7 ± 39.0) d	mild depression	(53.5 ± 16.9)/ (68.3 ± 7.4)/ (66.8 ± 17.2)	10 Hz rTMS/1 Hz rTMS/ Sham	L-DLPFC	80%	5 min/1 min; 1 s/10 s	450/900	20 min/d, 2–3 d/wk, 2 wk	BDI; MBI
Yang et al.(2014) [28]	China	37/37/37	28/9; 30/7; 27/10	-	-	-	moderate depression	(56.57 ± 13.62)/ (52.30 ± 11.03)/ (53.27 ± 14.65)	10 Hz rTMS/1 Hz rTMS/ Sham	L-DLPFC/ R-DLPFC	90%	5 s/35 s	1000	20 min/d, 5 d/wk, 4 wk	HAMD
Gu et al.(2017) [20]	Korea	12/12	6/6; 5/7	9/3; 8/4	-	(10.3 ± 2.7)/ (10.1 ± 2.3) m	mild depression	(58.1 ± 8.7)/ (58.3 ± 7.8)	10 Hz rTMS/ Sham	L-DLPFC	110%	5 s/1 min	1000	5 d/wk, 2 wk	HAMD; BDI
Kim et al. (2017) [19]	Korea	22/22	18/4; 13/9	11/11; 15/7	0/22; 0/22	-	severe depression	(52.6 ± 10.6)/ (64.3 ± 11.5)	5 Hz + 1 Hz rTMS/Sham	dual PL	90%	5 s/55 s + 5 min/1 min	-	20 min/d, 5 d/wk, 4 wk	BDI; FIM
Sasaki et al.(2017) [21]	Japan	7/6	5/2; 6/0	5/2; 2/4	4/3; 3/3	(4.1 ± 2.9)/ (5.3 ± 5.7) y	severe depression	(66.1 ± 11.2)/ (62.8 ± 10.1)	10 Hz rTMS/ Sham	DACC to MPFC	80%	10 s/50 s	2000	20 min/d, 5 d	QIDS
Zhang et al. (2017) [29]	China	48/47	28/20; 29/18	-	-	(32.64 ± 3.03)/ (32.41 ± 3.04) d	severe depression	(62.99 ± 6.05)/ (63.48 ± 5.91)	10 Hz rTMS/1 Hz rTMS	L-DLPFC/ R-DLPFC	90%	5 s/35 s;10 s/2 s	1500/1000	20 min/d, 5 d/wk, 4 wk	HAMD
Duan et al.(2018) [24]	China	32/32	14/18; 13/19	12/20; 10/22	15/17; 16/16	(49.53 ± 13.27)/ (50.19 ± 13.89) d	moderate depression	(71. 63 ± 8. 90)/ (71. 27 ± 7. 76)	1 Hz rTMS/ Sham	L-DLPFC	80%	-	1000	20 min/d, 6 d/wk, 4 wk	HAMD; MBI
Hu et al. (2020) [25]	China	14/13/15	10/4; 9/4; 9/6	9/5; 9/4; 11/4	-	(59.57 ± 49.28)/ (50.61 ± 48.38)/ (63.27 ± 47.67) d	moderate depression	(52.00 ± 9.64)/ (54.77 ± 11.31)/ (59.27 ± 8.58)	10 Hz rTMS/5 Hz rTMS/ Sham	L-DLPFC	100%	-	-	20 min/d, 5 d/wk, 3 wk	HAMD; MBI
Shan et al. (2022) [26]	China	40/39/37	23/17; 21/18; 19/18	30/10; 28/11; 29/8	20/20; 20/19; 21/16	(54.11 ± 7.69)/ (52.74 ± 6.40)/ (51.70 ± 6.55) d	moderate depression	(61.21 ± 3.6)/ (59.02 ± 3.1)/ (58.88 ± 4.3)	10 Hz + 1 Hz rTMS/ 10 Hz rTMS/ 1 Hz rTMS	dual DLPFC/ L-DLPFC/ R-DLPFC	80%	-	-	20 min/d, 5 d/wk, 6 wk	HAMD
Zeng et al.(2022) [22]	China	35/35	17/18; 19/16	-	-	(16.32 ± 4.71)/ (16.72 ± 4.28) w	severe depression	(63.7 ± 4.8)/ (62.6 ± 4.9)	iTBS/ 10 Hz rTMS	L-DLPFC	110%	-	2400	19 min/d; 33 min/d, 6 wk	HAMD
Dong et al.(2023) [23]	China	15/15	7/8; 6/9	10/5; 11/4	-	(1.44 ± 0.52)/ (1.50 ± 0.53) m	severe depression	(59.13 ± 6.74)/ (58.53 ± 5.94)	iTBS/ Sham	L-DLPFC	80%	2 s/8 s	1200	6 min 40 s, 3 wk	HAMD; MADRS
Yang et al.(2024) [27]	China	30/30	19/11; 17/13	-	13/17; 14/16	(5.87 ± 2.13)/ (5.63 ± 2.66) m	severe depression	(66.40 ± 6.62)/ (64.50 ± 6.68)	10 Hz rTMS/ Sham	L-DLPFC	100%	-	1200	5 d/wk, 4 wk	HAMD; BI

*E/C * experimental group/control group, *MT* motor threshold, *DLPFC* dorsolateral prefrontal cortex, *L-DLPFC* left DLPFC, *R-DLPFC* right DLPFC, *PL* parietal lobe, *DACC* dorsal anterior cingulate cortex, *MPFC* medial prefrontal cortex

### Study quality

**Fig. 2:**
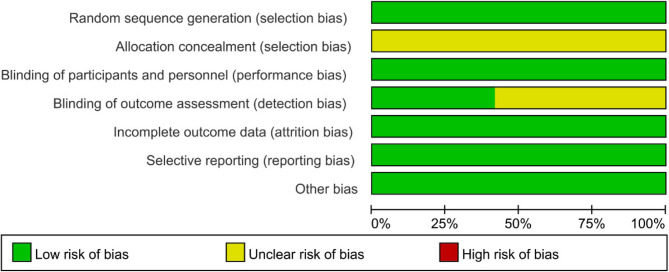
Risk of bias of the included studies

Eight of the 12 included studies [22–29] used random number tables to generate randomized sequences, and 4 studies [18–21] referred to “randomization” without describing the specific randomization method. None of the studies described allocation concealment. All studies were blinded to the subjects, and 5 studies [18, 20, 21, 24, 26] were blinded to the outcome assessors. All studies had complete outcome data and were free of selective reporting of findings and other biases (Fig. 2).

### Meta-analysis

#### The primary outcomes

**Fig. 3:**
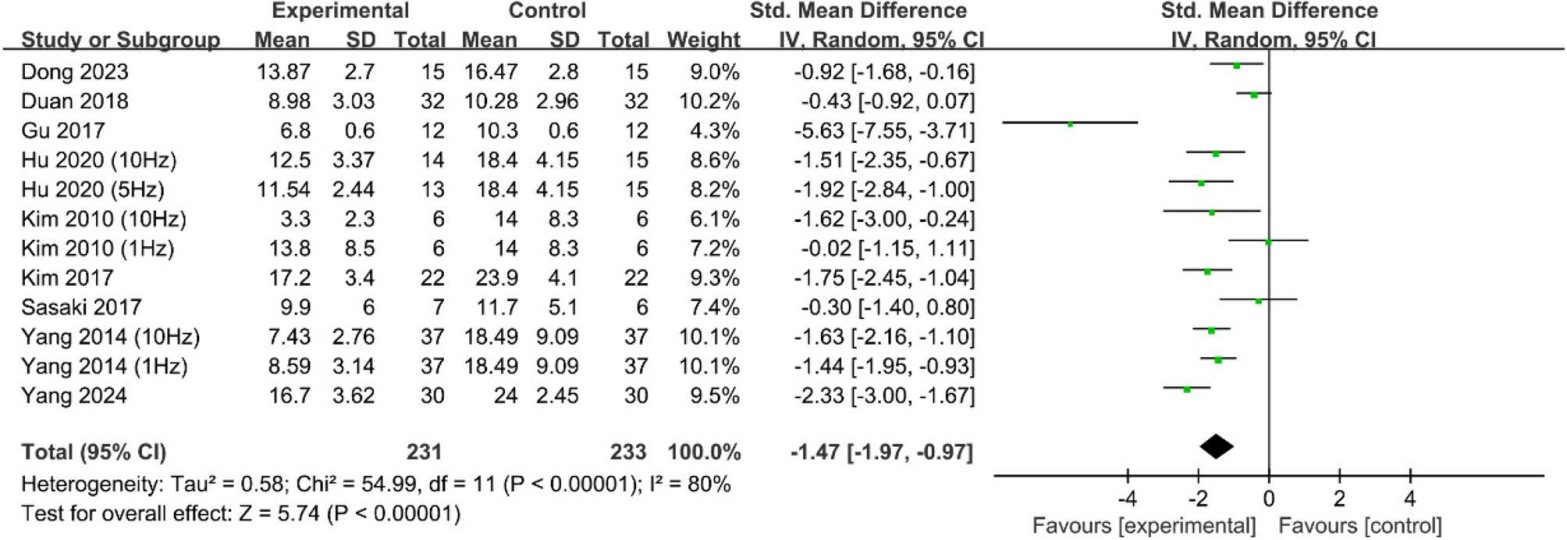
Meta-analysis of the effects of rTMS on depressive symptoms in PSD patients

Nine studies reported depression scores [18–21, 23–25, 27, 28], with significant heterogeneity among the studies (*P* < 0.1, *I*^2^ = 80%). The meta-analysis was performed via a random effects model. The results revealed that patients in the experimental group had significantly lower depression scores than did those in the control group (SMD = −1.47, 95% CI [−1.97, −0.97], *P* < 0.001; Fig. 3).

#### The secondary outcomes

**Fig. 4:**
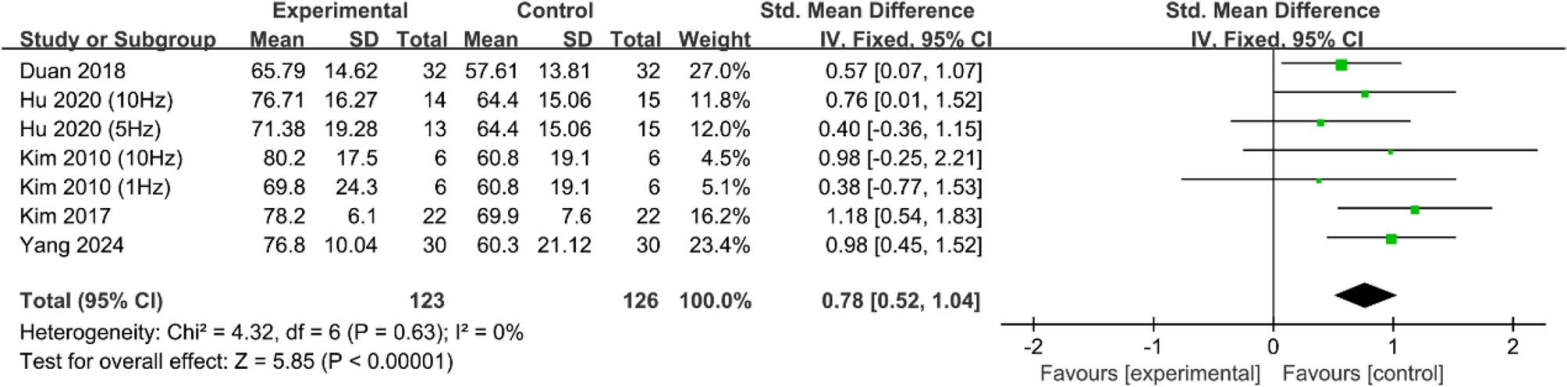
Meta-analysis of the effects of rTMS on activities of daily living in PSD patients

Activity of daily living (ADL) scores were reported in five studies [18, 19, 24, 25, 27]with no significant heterogeneity between studies (*P* > 0.1, *I*^2^ = 0%). The meta-analysis was performed using a fixed-effects model. The results revealed that patients in the experimental group had significantly higher ADL scores than did those in the control group (SMD = 0.78, 95% CI [0.52, 1.04], *P* < 0.001; Fig. 4).

#### Follow-up outcomes

**Fig. 5:**
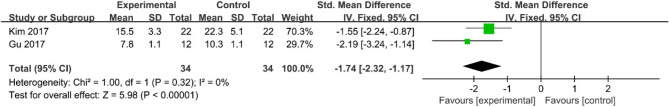
Meta-analysis of the effects of rTMS on follow-up in PSD patients at the end of treatment

Two studies [19, 20] reported depression scores at follow-up at the end of treatment, with no significant heterogeneity between studies (*P* > 0.1, *I*^2^ = 0%). The fixed-effects model was used for analysis. The results revealed that patients in the experimental group had significantly lower depression scores than did those in the control group (SMD = −1.74, 95% CI [−2.32, −1.17], *P* < 0.001; Fig. 5).

### Network meta-analysis

**Fig. 6:**
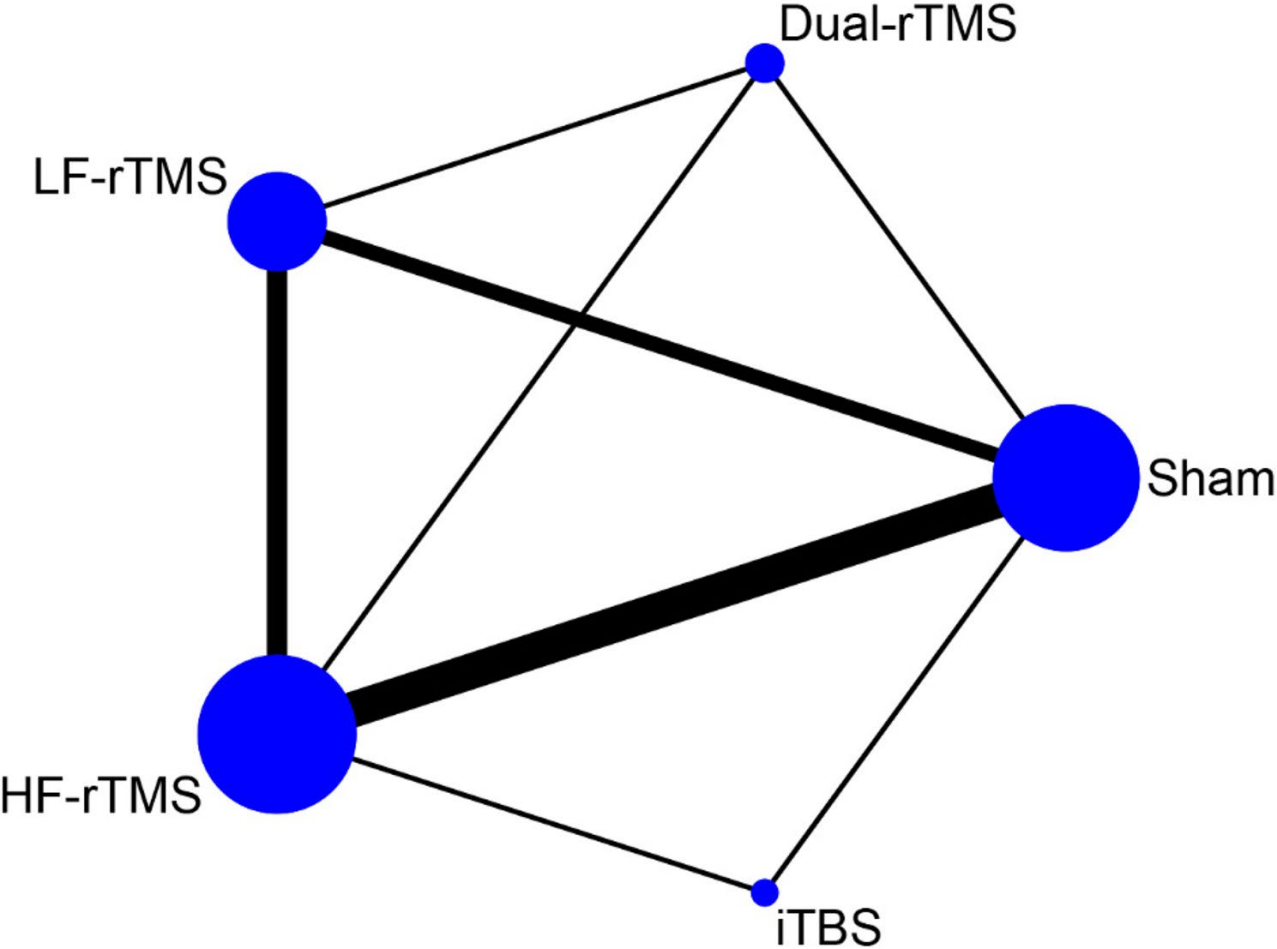
Network plot

**Fig. 7:**
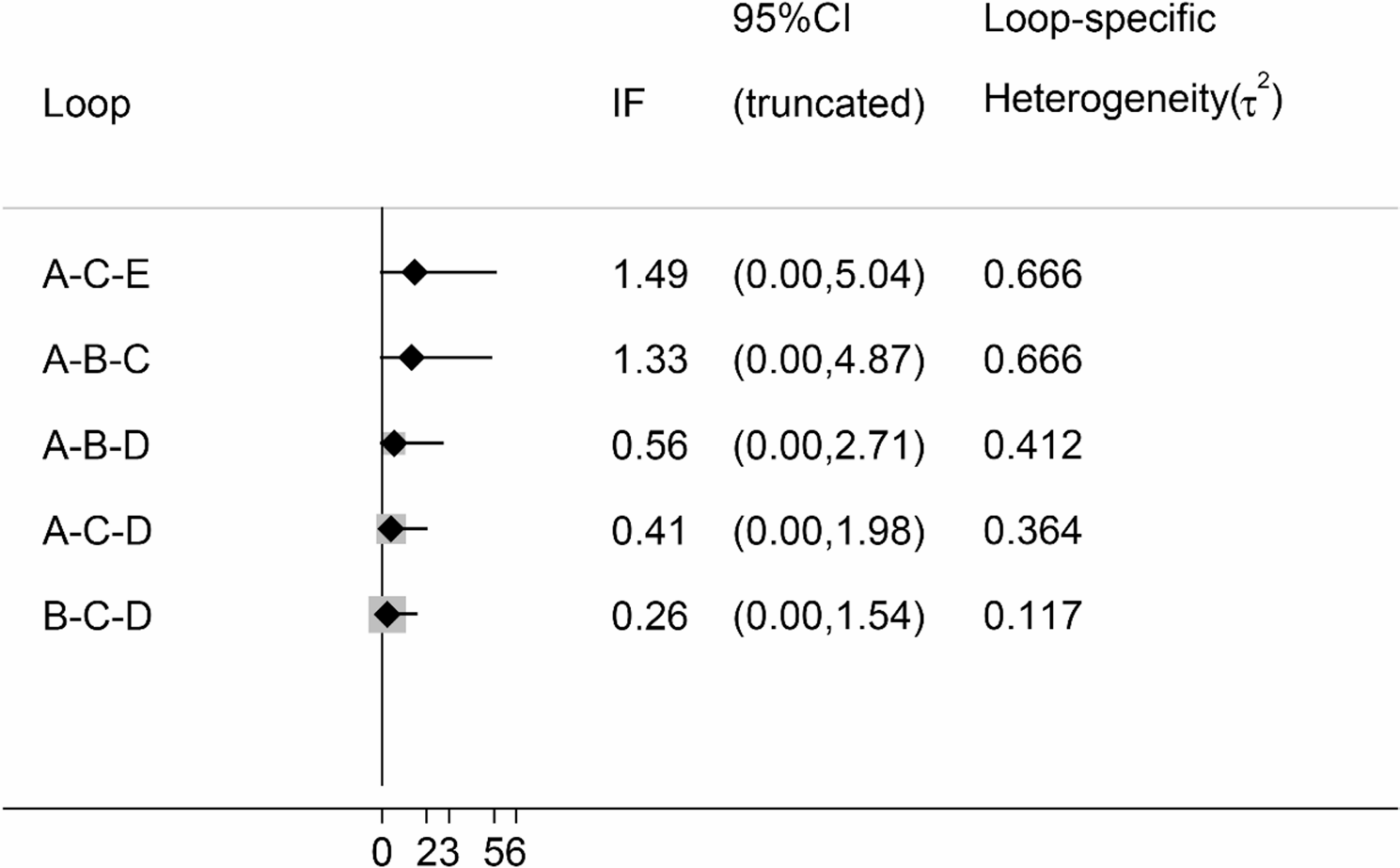
Inconsistency test. A, Sham; B, dual-rTMS; C, HF-rTMS; D, LF-rTMS; E, iTBS

**Fig. 8:**
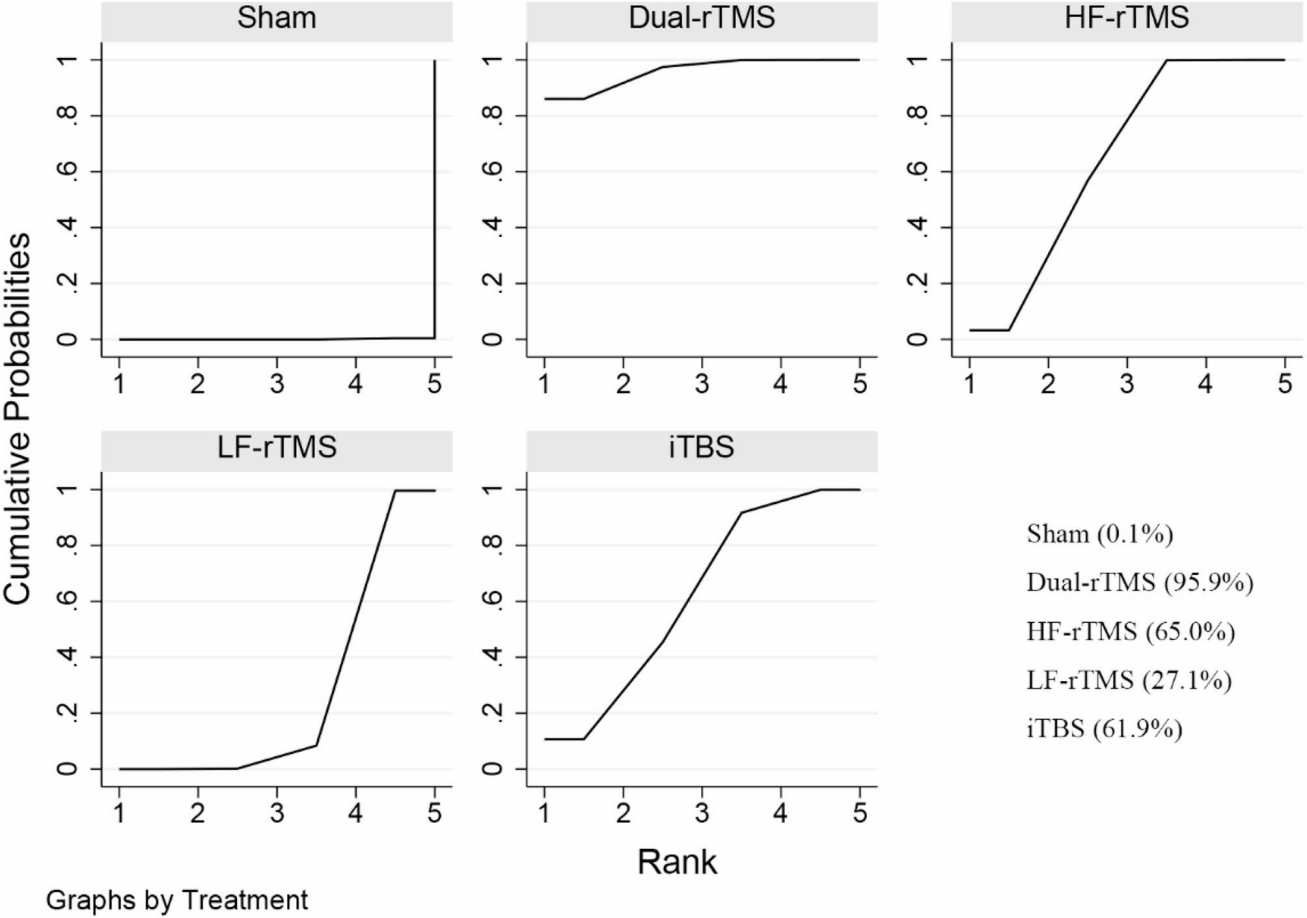
SUCRA plot

The network relationships between the different interventions, with depression scores as the outcome indicator, are shown in Fig. 6. The results of the inconsistency test revealed that the 95% CI of each closed loop was 0 and *P* > 0.05, indicating that the consistency of each closed loop was good; thus, the consistency model was used in this study (Fig. 7). Pairwise comparisons of all interventions are shown in the league table in Table 2. Compared with sham stimulation, dual-rTMS (SMD = −2.33, 95% CI [−3.16, −1.50]), HF-rTMS (SMD = −1.62, 95% CI [−2.11, −1.13]), iTBS (SMD = −1.57, 95% CI [−2.56, −0.57]), and LF-rTMS (SMD = −0.80, 95% CI [−1.36, −0.23]) significantly improved depression. The SUCRA values of the different interventions for reducing depression scores were in the following order: dual-rTMS (95.9%) > HF-rTMS (65.0%) > iTBS (61.9%) > LF-rTMS (27.1%) (Fig. 8).

**Table 2:** League table for the changes in depression. The results labelled in bold indicate statistical significance

dual-rTMS	0.71 (-0.11,1.53)	1.54 (0.70,2.37)	0.76 (-0.48,2.00)	2.33 (1.50,3.16)
-0.71 (-1.53,0.11)	HF-rTMS	0.83 (0.28,1.38)	0.06 (-0.93,1.04)	1.62 (1.13,2.11)
**-1.54 (-2.37, -0.70)**	**-0.83 (-1.38, -0.28)**	LF-rTMS	-0.77 (-1.85,0.31)	0.80 (0.23,1.36)
-0.76 (-2.00,0.48)	-0.06 (-1.04,0.93)	0.77 (-0.31,1.85)	iTBS	1.57 (0.57,2.56)
**-2.33 (-3.16, -1.50)**	**-1.62 (-2.11, -1.13)**	**-0.80 (-1.36, -0.23)**	**-1.57 (-2.56, -0.57)**	Sham

### Adverse events

Among the 12 studies, 8 [18, 20–23, 25, 26, 28] reported adverse effects of rTMS, among which 5 [18, 20–22, 28] reported that patients did not experience any adverse effects or complications during the treatment process. Three [23, 25, 26] studies reported that 1 or 2 patients experienced a transient mild headache, which was relieved after resting and gradually adapting to the treatment, and none of the patients experienced any other adverse effects; thus, rTMS generally exhibited good safety.

### Subgroup analysis of the primary outcomes

The depression scores of treated PSD patients were analysed in subgroups based on the frequency of rTMS stimulation, MT, and patients’ level of depression. The results are shown in Table 3.

**Table 3:** Subgroup analysis of depression in patients with PSD after treatment

Subgroup analysis		Studies	SMD (95% CI)	*P*	X^2^	I^2^ (%)	Tau^2^
Stimulation frequency	HF-rTMS	7	−1.73 [−2.54, −0.92]	< 0.0001	34.77	83%	0.92
	LF-rTMS	3	−0.71 [−1.55, 0.12]	0.09	9.89	80%	0.41
	dual-rTMS	1	−1.75 [−2.45, −1.04]	< 0.0001	-	-	-
	iTBS	1	−0.92 [−1.68, −0.16]	0.02	-	-	-
Motor threshold	80%	5	−0.57 [−0.97, −0.18]	0.004	4.49	11%	0.02
	90%	3	−1.58 [−1.91, −1.25]	< 0.0001	0.53	0%	0.00
	100%	3	−1.98 [−2.47, −1.49]	< 0.0001	2.28	12%	0.02
	110%	1	−5.63 [−7.55, −3.71]	< 0.0001	-	-	-
Depression level	mild	3	−2.33 [−5.24, 0.58]	0.12	24.42	92%	6.02
	moderate	5	−1.34 [−1.89, −0.79]	< 0.0001	15.44	74%	0.28
	severe	4	−1.39 [−2.22, −0.57]	0.0009	13.24	77%	0.54

### Sensitivity analysis

Sensitivity analysis of the meta-analysis results by the one-by-one exclusion method revealed that the combined effect values did not change significantly, suggesting that the study results were more stable.

### Publication bias

**Fig. 9:**
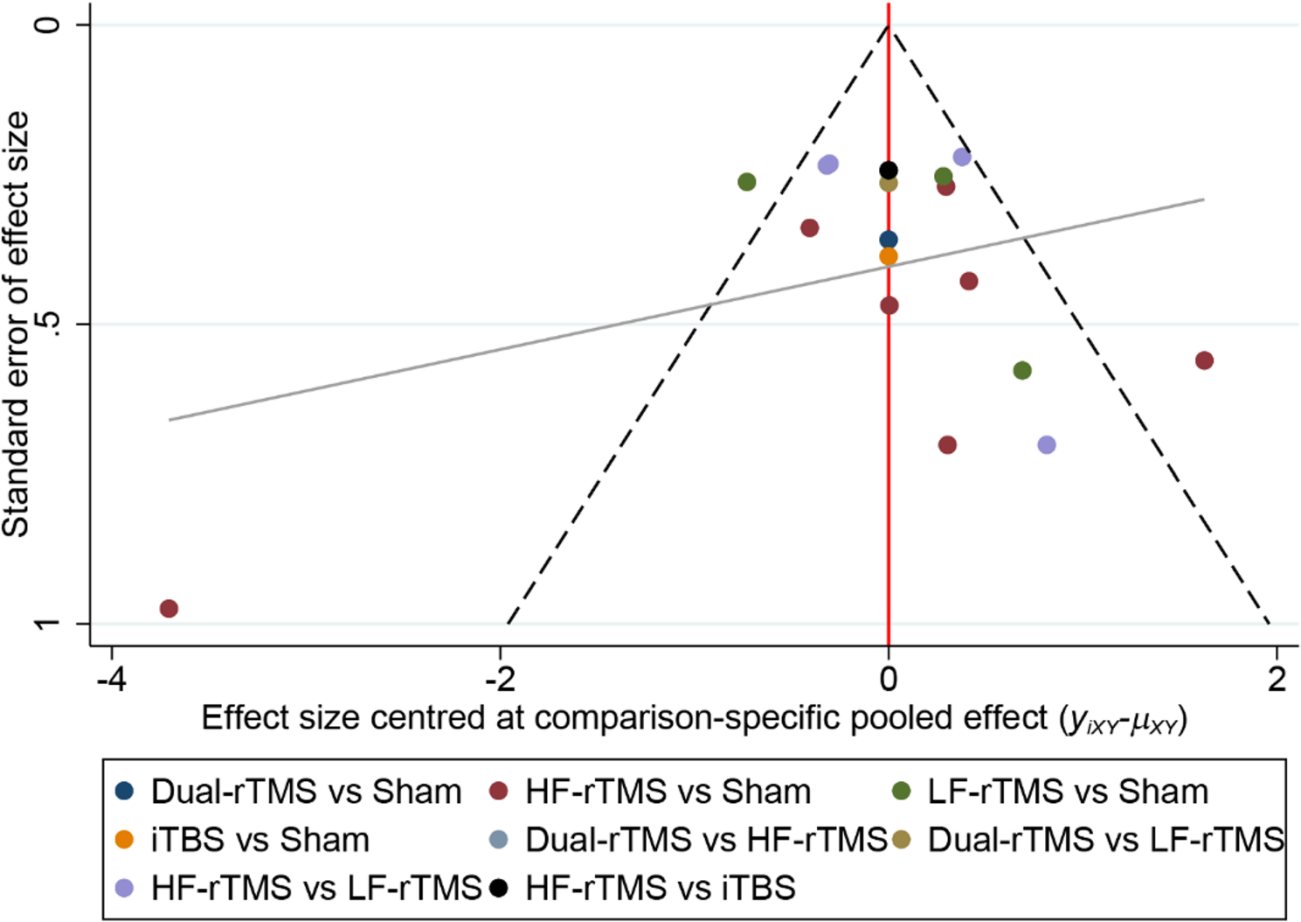
Comparison-corrected funnel plot (*P* = 0.514)

The symmetry of the comparison-correction funnel plot was fair, and most of the scatter was located in the middle and upper parts of the triangle, but three studies fell outside the 95% CI (Fig. 9). Egger’s test revealed no significant bias in the results of the network meta-analysis (*P* > 0.1).

## Discussion

This study included a total of 12 RCTs, and the results of the meta-analysis revealed that dual-rTMS, HF-rTMS, LF-rTMS, and iTBS improved depression and ADL in patients with PSD. The results of the network meta-analysis suggest that dual-rTMS may be more effective than other interventions in achieving antidepressant effects in patients with PSD. This result is consistent with the findings of Sun et al., who reported that dual-rTMS and HF-rTMS were the most prioritized noninvasive brain stimulation interventions to improve PSD [30]. However, our findings regarding the improvement in depressive symptoms with dual-rTMS therapy were primarily derived from two RCTs. Therefore, caution should be taken in interpreting the findings to ensure that further clinical trials provide additional evidence. The DLPFC is the main target of rTMS for the treatment of depression, and the use of dual-rTMS may produce a more favourable short-term antidepressant effect than rTMS, which stimulates only one side of the brain region in patients with PSD. In the included studies, the duration of rTMS was mostly 20 min, once a day, 5 days a week, for a total intervention time of approximately 4 wk.

PSD occurs as a result of post-stroke damage to certain amine transmitter-related sites in the brain, including the brainstem (especially the midbrain superior projection fibres), thalamus, basal ganglia, and frontal cortex [31]. The neurobiological basis of PSD is primarily an imbalance in the 5-HT, norepinephrine (NE), and dopamine (DA) systems [32, 33]. 5-HT, an inhibitory neurotransmitter, plays an important role in regulating sleep and controlling mood, learning, and memory [34]. When the body is under stress, the expression of 5-HT and its receptor is abnormal, or its function is decreased, which contributes to the occurrence of mood disorders. Studies have shown that rTMS has definite efficacy in treating depression and anxiety-affective disorders. The main therapeutic mechanism of rTMS is that rTMS can effectively promote the release of more neurotransmitters, such as 5-HT and dopamine, from the striatum and hippocampus of PSD patients, regulate the excitability of the bilateral cerebral cortex, increase local cerebral blood flow, and improve metabolism in brain areas [27, 35, 36]. rTMS may treat PSD by modulating the levels of BDNF. BDNF is an important neurotrophic factor that is widely distributed in the central nervous system and is essential for the survival, growth, and maintenance of neurons in brain regions associated with mood and cognitive function [37]. rTMS enhances the expression of BDNF by activating the Ca2+-CaMKII-CREB pathway in Neuro-2a cells [38].

The interhemispheric inhibition model refers to the existence of inhibition of one cerebral hemisphere to the other cerebral hemisphere. In healthy people, the inhibition between the two cerebral hemispheres is transmitted through the nerve fibres in the corpus callosum, which can balance the functions of the two hemispheres [39]. After stroke, the affected hemisphere is unable to undergo normal inhibition of the healthy hemisphere, resulting in pathologic excitation of the healthy hemisphere, which leads to depression [40]. Therefore, HF-rTMS is commonly used to stimulate the motor cortex of the affected hemisphere, and LF-rTMS is commonly used to inhibit the motor cortex of the healthy hemisphere to promote the recovery of patients [41, 42]. Dual-rTMS enhances the excitability of the cortex of one hemisphere while decreasing the excitability of the cortex of the other hemisphere, which is a potential reason why bilateral rTMS seems to be more effective than unilateral rTMS.

The following limitations exist in this study: (1) owing to the nature of the studies, it was difficult to implement blinding, the quality of the included literature was low, and implementation bias may have existed; (2) a limited number of original studies related to some of the interventions were included, and their small sample sizes led to uncertainty in the results generated in this study; (3) because of the variability of rTMS regimens and the lack of data on long-term follow-up, it is not possible to accurately compare the efficacy and long-term outcomes of various rTMS treatments. In short, the above factors should be fully considered when applying the evidence from this study, and they should be used with caution.

## Conclusions

This study is the first to evaluate the efficacy of different modes of rTMS for PSD. It was found that 4wk of 20-min daily dual-rTMS on the DLPFC of PSD patients may produce better therapeutic effects. These findings may provide some reference data for the selection of rTMS stimulation programs in clinical practice. In the future, high-quality, large-sample, standardized RCTs of different modalities of rTMS could be conducted. The scientific validity of the findings can be further tested to determine the best treatment options for rTMS and improve its efficacy.

## Supplementary Information

Supplementary Material 1.

## Data Availability

No datasets were generated or analysed during the current study.

## Abbreviations

PSD: Post-stroke depression
rTMS: Repetitive transcranial magnetic stimulation
HF-rTMS: High frequency-rTMS
LF-rTMS: Low frequency-rTMS
TBS: Theta-burst stimulation
PRISMA: Preferred Reporting Items for Systematic Review and Meta-Analysis
RCT: Randomized controlled trials
HAMD, HDRS: Hamilton Depression Rating Scale
BDI: Beck Depression Inventory
QIDS: Quick Inventory of Depressive Symptomatology
MADRS: Montgomery Asberg Depression Rating Scale
MBI: Modified Barthel Index
BI: Barthel Index
FIM: Functional Independence Measure
